# PREOPERATIVE PHYSIOTHERAPY AND ONE-YEAR PATIENT-REPORTED OUTCOMES AFTER PRIMARY TOTAL KNEE ARTHROPLASTY: A REGISTRY-BASED COHORT STUDY OF 1,688 PATIENTS

**DOI:** 10.2340/jrm.v58.44853

**Published:** 2026-07-02

**Authors:** Siri B. WINTHER, Mani IZADI, Vigdis K. S. HUSBY, Otto S. HUSBY, Mathias F. BROBAKKEN, Frank-David ØHRN, Jomar KLAKSVIK, Eivind WANG, Ole Kristian BERG

**Affiliations:** 1 Department of Orthopaedic Surgery, Clinic of Orthopedics, Rheumatology and Dermatology, St. Olav’s University Hospital HF, Trondheim; 2 Department of Neuromedicine and Movement Science, Faculty of Medicine and Health Science, Norwegian University of Science and Technology NTNU, Trondheim; 3 Faculty of Health Sciences and Social Care, Molde University College, Molde; 4 Department of Health Sciences Aalesund, Faculty of Medicine and Health Science, Norwegian University of Science and Technology, Aalesund; 5 Orthopaedic Department, Nordmøre and Romsdal Hospital, Møre and Romsdal Hospital Trust, Norway

**Keywords:** preoperative physiotherapy, total knee arthroplasty, physical function, prehabilitation

## Abstract

**Objective:**

Preoperative physiotherapy in patients receiving primary total knee arthroplasty (TKA) aims to relieve pain, delay surgery, and improve postoperative recovery. This study investigates the change in PROMs and pain after primary TKA between patients who received preoperative physiotherapy (P) and those who did not (NP).

**Design:**

Registry-based cohort study with data from an institutional registry.

**Patients:**

1,688 patients followed a standardized fast-track clinical pathway between August 2017 and January 2024 and were grouped in P or NP.

**Methods:**

Primary outcome was KOOS-PS at 2 months and 1 year postoperatively. Secondary outcomes included pain, the Forgotten Joint Score, and EQ-5d-5L. Two anchor questions related to self-perceived knee function and willingness to have the surgery again at 12 months’ follow-up were also evaluated.

**Results:**

The model estimate demonstrated no significant between-group difference in KOOS-PS at 2 months (1.12 points; *p* = 0.079) or 1-year follow-up (1.25 points; *p* = 0.097). Visual inspection of descriptive plots showed that NP patients had higher KOOS-PS, less pain, and better joint score and quality of life at all time points. At 12 months’ follow-up, both groups had similar responses to the anchor questions.

**Conclusion:**

After adjustment for baseline differences, no between-group differences in postoperative self-reported physical function were observed; consistently lower scores in the physiotherapy group may reflect systematic preoperative differences between groups.

## LAY ABSTRACT

Before knee replacement surgery, some patients undergo physiotherapy to manage pain and prepare for recovery. This study examined whether preoperative physiotherapy influenced outcomes after surgery. We followed 1,688 patients and compared those who received physiotherapy beforehand with those who did not. Over the following year, both groups showed improvements in pain, physical function, and quality of life. Interestingly, patients who did not receive physiotherapy started with slightly better scores and continued to report less pain and better joint function throughout the year. However, by the end of the year, satisfaction levels were similar across both groups. These findings suggest that there may be differences in the types of patients who choose preoperative physiotherapy vs those who do not. While physiotherapy may benefit some individuals before surgery, it might not be necessary for everyone. This insight can help healthcare professionals tailor pre-surgical treatment plans to better meet individual patient needs.

A recent review from 2024 reports more than 360 million prevalent cases of knee osteoarthritis (OA) worldwide (1). The economic burden of the disease encompasses both direct healthcare costs related to treatment and surgery, as well as indirect costs arising from disability, reduced productivity, and the need for longterm care, accounting for approximately 1.0–2.5% of GDP in developed countries (2). As global populations age and life expectancy continues to increase, the prevalence of osteoarthritis (OA) is rising. Consequently, the number of patients undergoing total knee arthroplasty (TKA) is expected to increase substantially, reaching approximately 1.26 million procedures annually by 2030 (3). Patients with knee OA often become inactive prior to surgery due to activity-related pain. This inactivity in turn leads to a loss of muscle mass, mobility, and function, which becomes a vicious circle of constant deconditioning (4). Often, activity levels become reduced due to pain during weightbearing and movement. Physical inactivity accelerates loss of muscle strength, joint mobility, and overall physical function, thereby exacerbating pain and functional limitations. As symptoms progress, patients further limit activity, reinforcing a selfperpetuating cycle of deconditioning that diminishes functional capacity and surgical readiness (5).

Prehabilitation aims to enhance a patient’s functional fitness before surgery, minimizing the decline in function. It involves interventions delivered in the preoperative period to optimize a patient’s physical and psychological capacity for surgery and improve postoperative outcomes (6). It may include exercise training with or without supervised physiotherapy, nutritional optimization, psychological support, patient education, and lifestyle modifications, delivered as singlecomponent or multimodal programmes (7). It has been found to reduce the length of hospital stay and decrease the convalescence period after TKA (8–11). This might reduce the risk of further reduction in function and postoperative complications (12).

Reviews have reported low to moderate evidence that preoperative physiotherapy interventions reduce pain and improve functional performance in patients with knee osteoarthritis (OA) (13, 14). One review found that preoperative exercise interventions prior to TKA reduced pain and improved knee function, muscle strength, and quality of life (15). Furthermore, studies focusing on preoperative high-intensity strength exercises have demonstrated superior functional performance, greater strength, and improved PROMs following TKA (10, 16–18). However, other studies have found no advantage of prehabilitation following TKA (19–23), and a review concludes that there is no evidence that prehabilitation provides benefits in terms of function, pain, or quality of life in patients with arthroplasty for osteoarthritis (21). Consequently, there is no clear consensus in the literature on whether prehabilitation has any effect, likely due to the significant variation in content and type (9, 24). Several reviews and meta-analyses have been published on this topic, but the heterogeneity of the studies makes direct comparisons challenging (11, 24). Heterogeneity with respect to study design, patient populations, interventions, and outcome measures complicates direct comparisons and limits data pooling in such studies (24). There is a lack of large registry studies evaluating the results of preoperative physiotherapy, specifically on pre- and postoperative PROMs. Registrybased studies include large, unselected patient populations treated in routine clinical practice, thereby enhancing external validity and generalizability. While RCTs remain essential for establishing causal effects under controlled conditions, registrybased studies provide complementary realworld evidence on effectiveness, safety, and implementation in everyday clinical settings (25, 26).

As the prevalence of osteoarthritis rises, indiscriminate use of preoperative physiotherapy for all TKA candidates risks imposing a substantial economic strain if meaningful clinical benefit is absent. Inconsistent functional outcomes and equivocal evidence underscore uncertainty regarding both which patients derive benefit and which interventions are effective. From a valuebased care perspective, widespread implementation is difficult to justify unless prehabilitation is targeted to patients most likely to benefit and confined to interventions associated with clinically meaningful effects (27).

Inconclusive evidence for the benefit of preoperative physiotherapy and the lack of registry studies evaluating preoperative physiotherapy in arthroplasty candidates provide a rationale for the present registrybased analysis of realworld effectiveness. The aim of this study was to evaluate PROMs and pain at 2 months and 1 year postoperatively in patients undergoing primary TKA within a standardized fasttrack care pathway. The comparison was made between patients who received preoperative physiotherapy with those who did not. Physical function, assessed by the KOOSPS, was defined as the primary outcome.

## METHODS

The study was approved by the Regional Committee for Medical and Health Research Ethics (REC central) (approval no. 522685) and conducted in accordance with the Helsinki Declaration. Patients were informed about the registry and gave written informed consent to allow data to be used for scientific purposes before inclusion. This study conforms to all STROBE guidelines and reports the required information accordingly.

### Design

This study is a registry-based cohort study with data from an institutional registry for patients undergoing TKA. Patients were informed about the registry and gave written informed consent to allow data to be used for scientific purposes before inclusion. All patients were treated according to a previously described standardized fast-track clinical pathway (28), also known as enhanced recovery after surgery (ERAS). It is a coordinated, multimodal, evidencebased perioperative care programme designed to optimize recovery, reduce complications, shorten length of hospital stay, and improve patient satisfaction after hip or knee replacement. These pathways integrate improvements across the entire surgical journey – before, during, and after surgery (29).

### Patients

**Fig. 1:**
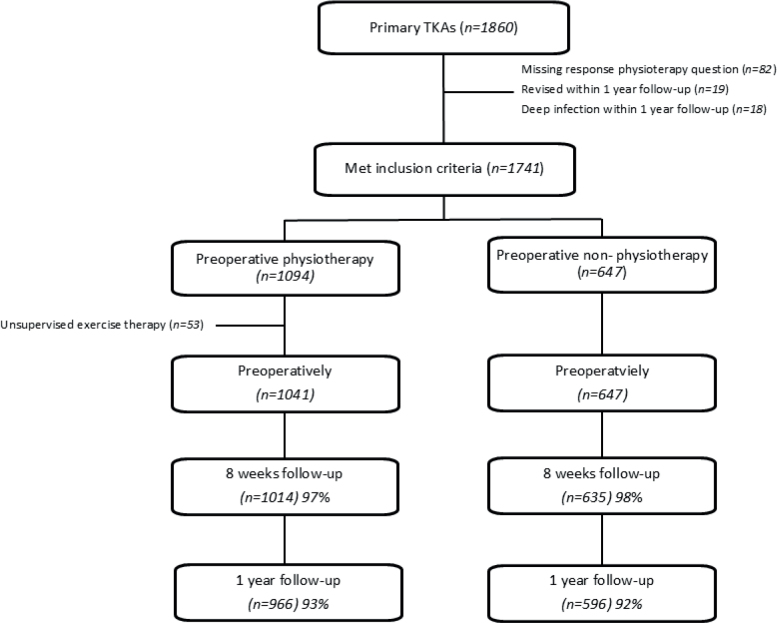
Study flowchart.

All patients undergoing elective primary TKA performed at St. Olav’s University Hospital from August 2017 until January 2024 were eligible for study inclusion. Exclusion criteria were missing data related to preoperative physiotherapy, revision for any cause, and/or deep infection within 12 months’ follow-up. The patients were retrospectively grouped into 2 categories based on whether they had supervised preoperative physiotherapy (P) or not (NP). Patients who had had preoperative physiotherapy consisting of unsupervised exercise therapy were also excluded, leaving only those with supervised exercise therapy in the preoperative physiotherapy group (Fig. 1). Patient characteristics are given in Table I.

**Table I:** Patient demographics and clinical outcomes

Factor	Preoperative physiotherapy (*n* = 1,041)	Preoperative non-physiotherapy (*n* = 647)	Total (*n* = 1,688)
Age (range)	67 (36–92)	69 (36–90)	68 (36–92)
0BMI (range)	30 (15–46)	29 (14–46)	30 (14–46)
Sex (% females)	70	53	64
ASA (% I/II/III/IV)	9/67/24/1	9/60/31/1	9/64/27/1
LOS days (range)	2.2 (0–14)	2.1 (0–12)	2.2 (0–14)
Preop. walking aid (%)			
Crutches/walking frame	13	8	11
Two walking sticks	10	6	8
One walking stick	11	15	13
Complications			
Mechanical	0	1 (0.1%)	1 (0.1%)
Stiffness	57 (5.5%)	26 (4.0%)	84 (5%)
DVT	7 (0.7%)	3 (4.6%)	10 (0.6%)
Readmission	18 (1.7%)	10 (1.5%)	28 (1.7%)
Reoperation	24 (2.3%)	12 (1.9%)	36 (2.1%)

Values are presented as *n* or mean. ASA: American Society of Anesthesiologists physical status, BMI: body mass index, LOS: length of hospital stay, DVT: deep vein thrombosis, TKA: total knee arthroplasty. Each surgery could be registered with several complications. Readmissions: readmitted without any reoperation, reoperation: surgery without any revision of components within 12 months’ follow-up.

### Preoperative physiotherapy

Preoperative physiotherapy does not have any universally recognized standard. Studies on preoperative physiotherapy describe the frequency for most knee osteoarthritis patients to be once (58%) or twice (41%) per week, for less than 4 weeks, with the main mode being individual sessions lasting between 20 and 60 min (30, 31). With more than 97% of sessions containing active strengthening, aerobic, gait, balance, or range of motion exercises with a high number of repetitions with low or no external load. In addition, more than 80% of the sessions including functional exercises such as stair climbing or chair rising (30). This description aligns well with findings from studies in Norway, where the current investigation was conducted (32). Patients in the present study were asked at the preoperative outpatient clinic if they had supervised physiotherapy.

### Data management

Data were registered by nurses, physiotherapists, and by the patients. Data collection was obtained at the preoperative outpatient clinic, during hospitalization, and at 2 and 12 months’ follow-up.

### Outcomes

The primary outcome was the disease-specific Knee Injury and Osteoarthritis Outcome Score – Physical Function – Short Form (KOOS-PS) at 2 months and 1 year postoperatively. The questionnaire assessed physical function and ranges from 0 to 100, the latter representing no difficulty in performing different tasks.

Secondary outcomes were health-related quality of life assessed by the generic European Quality of Life – 5 Dimension questionnaire (EQ-5D-5L), ranging from −0.59 to 1.00, where 1.00 represents perfect health and values less than 0 represents a health state that is “worse than death”. The Forgotten Joint Score (FJS), a 12-item questionnaire detecting the patient’s awareness of the artificial joint during daily life activities. The score ranges from 0–100 where higher scores are associated with a higher degree of “forgetting” the joint. Pain during mobilization and at rest was reported by the numeric rating scale (NRS 0–10) (0 representing no pain). At 12 months’ follow-up, self-perceived function and willingness to undergo the surgery again was evaluated by 2 anchor questions: (*i*) “How does the leg that was operated on work today compared to before surgery?” (“Better”, “Same”, “Unable to discriminate”, or “Worse”), and (*ii*) “Based on your experience to date, would you go through the surgery again?” (“Yes”, “No”, or “Unable to decide”). Length of hospital stay, postoperative complications, readmissions, and reoperations were also registered.

### Statistics

A linear mixed model (LMM) was used to analyse the primary outcome KOOS-PS at 2 and 12 months between the groups. Groups and time points were modelled as fixed factors. Age, sex, baseline walking ability, and preoperative KOOS-PS score were used as covariates in the analysis. A random subject intercept was included. Normality of residuals was verified by histogram. Figures and numbers in the text are based on descriptive mean values with 95% confidence intervals (CI). Non-overlapping confidence intervals were interpreted as a statistically significant difference. Statistical analyses were performed using the software package IBM SPSS Statistics for Windows, Version 29 (IBM Corp, Armonk, NY, USA).

## RESULTS

A total of 1,688 cases were included, of which 1,094 (62%) had preoperative physiotherapy (see Fig. 1). Baseline patient characteristics were similar in both groups except for sex and walking aid. There were 70% (727) women in the P group and 53% (340) in the NP group. Also, 136 (13%) patients in the P group used crutches or a walking frame, whereas in the NP group the number was 54 (8%) (see Table I).

### Primary outcome

**Fig. 2:**
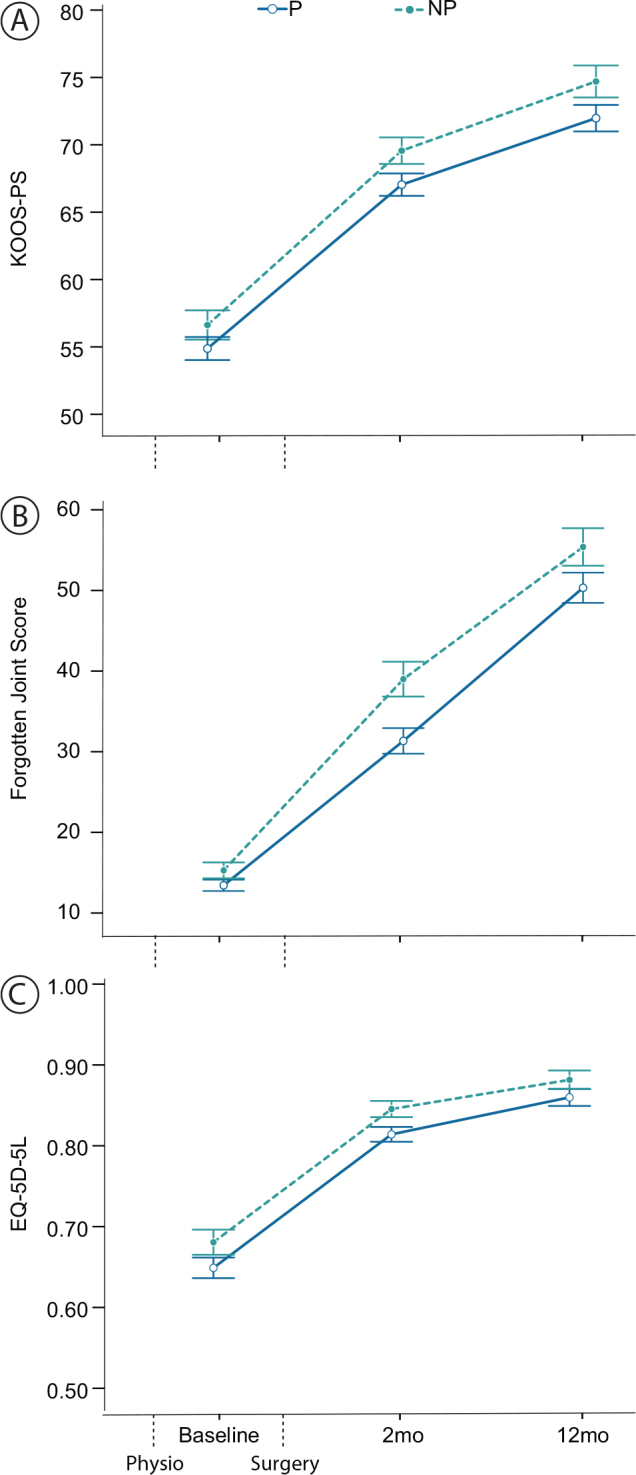
Mean and CI of (A) Knee Injury and Osteoarthritis Outcome Score – Short form; (B) the Forgotten Joint Score, and (C) EQ-5D-5L preoperatively, at 2 months and 1-year follow-up in the preoperative physiotherapy (P) and preoperative non-physiotherapy (NP) groups.

Model estimates of KOOS-PS in the P and NP groups were mean 67.9 (95% CI 67.1, 68.7) and 69.0 (CI 68.9, 69.1) at 2 months, and 72.7 (CI 71.8, 73.6) and 74.0 (CI 72.8, 75.2) at 1 year follow-up, respectively. No differences in KOOS-PS at 2 months (1.12 points; *p* = 0.079) or 1-year follow-up (1.25 points; *p* = 0.097) between the groups were found (*p* > 0.079) (Fig. 2A).

### Secondary outcomes

FJS in the P group was 13.4 (12.7, 14.1) preoperatively and increased to 31.3 (29.7, 33.0) at 2 months, and 50.3 (48.5, 52.2) at 1 year follow-up. Corresponding results in the NP group were 15.3 (14.3, 16.3), 39.0 (36.8, 41.1), and 55.4 (53.1, 57.7) respectively (Fig. 2B).

EQ-5D in the P group was 0.65 (0.64, 0.66) preoperatively and increased to 0.81 (0.80, 0.82) at 2 months, and 0.86 (0.85, 0.87) at 1 year follow-up. Corresponding results in the NP group were 0.68 (0.66, 0.70), 0.85 (0.84, 0.86), and 0.88 (0.87, 0.89) respectively (Fig. 2C).

**Fig. 3:**
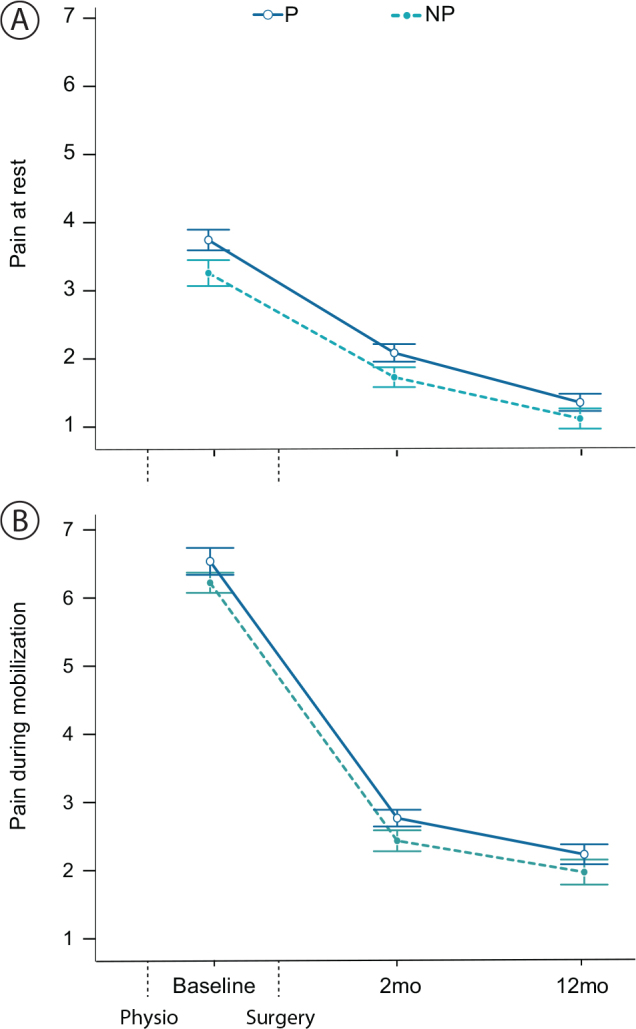
Mean and CI of pain (A) at rest and (B) during mobilization preoperatively, at 2 months and 1 year follow-up in the preoperative physiotherapy (P) and preoperative non-physiotherapy (NP) groups.

Pain at rest in the P group was 3.7 (3.6, 3.9) preoperatively and decreased to 2.1 (2.0, 2.2) at 2 months, and 1.4 (1.2, 1.5) at 1 year follow-up. Corresponding results in the NP group were 3.3 (3.1, 3.4), 1.7 (1.6, 1.9), and 1.1 (1.0, 1,3) respectively (Fig. 3A).

Pain during mobilization in the P group was 6.5 (6.3, 6.7) preoperatively and decreased to 2.8 (2.7, 2,9) at 2 months, and 2.2 (2.1, 2.4) at 1 year follow-up. Corresponding results in the NP group were 6.2 (6.1, 6.4), 2.4 (2.3, 2.6), and 2.0 (1.8, 2.2) respectively (Fig. 3B).

Overall, based on visual inspection of descriptive plots we found that patients who did not attend preoperative physiotherapy had higher KOOS-PS, less pain at rest and during mobilization, and better joint score and quality of life at all time points (see Figs 2 and 3).

**Fig. 4:**
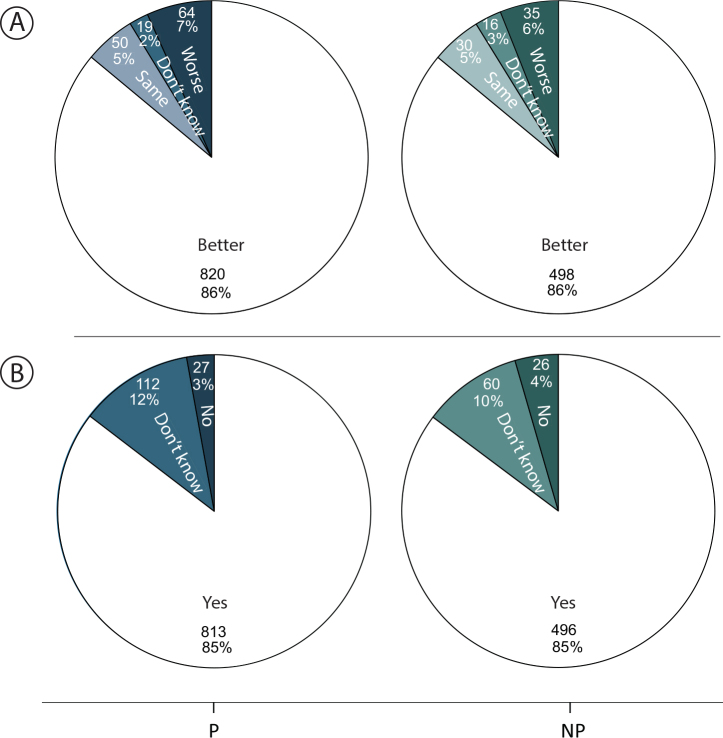
(A) Self-perceived knee function and (B) willingness to have the surgery again in the preoperative physiotherapy (P) and preoperative non-physiotherapy (NP) groups at 1-year follow-up.

At 12 months’ follow-up 86% reported improved function in the knee compared with before surgery in both groups. Similarly, 85% responded that they would have had the surgery again given the current knowledge (Fig. 4). Length of hospital stay, complications, readmissions, and reoperations were similar in both groups (Table I).

## DISCUSSION

This study examined PROMs and pain following TKA to evaluate whether patients did benefit from preoperative physiotherapy in the postoperative rehabilitation process. Conversely, the present findings demonstrated similar recovery over time for both groups. There were no differences between the groups in physical function measured by KOOS-PS at 2 months’ or 1 year follow-up when adjusting for covariates. The KOOS-PS increased similarly in both groups to 12 months’ follow-up, with the NP group consistently showing higher scores at all time points (see Fig. 2A), but the difference was far from the 5.1 points considered clinically relevant (33). Also, the NP group consistently reported better descriptive PROMs such as FJS and EQ-5D, and pain at all time points (see Figs 2 and 3).

In Norway, access to physiotherapy is considered an integral part of the public healthcare system, and the state plays a major role in financing these services. Patient-related physiotherapy is partially subsidized by the government, which significantly reduces out-of-pocket expenses. This makes physiotherapy broadly accessible, independent of patients’ socioeconomic status. Consequently, the societal costs are considerable, and allocating resources to such interventions would be unjustified in the absence of added benefit.

The findings of the present study are supported by the literature in which recent systematic reviews and meta-analysis suggest that preoperative physiotherapy has minimal or no clinically meaningful effect on postoperative pain and function following TKA (9, 13, 23, 34–36). A recent umbrella review concluded that preoperative exercise and physiotherapy have negligible effects on postoperative pain and function but could reduce hospital length of stay (34). Another large meta-analysis found that prehabilitation might improve pain and function during the early postoperative period, concluding that these effects were small, short-lived, and did not impact quality of life or overall healthcare costs (35).

Pain at rest and during mobilization was higher in the P group at all time points. However, the observed pain differences in our study were minor and not considered clinically relevant (37). These results are supported by studies showing no advantage of prehabilitation on postoperative pain beyond 3 months postoperatively compared with no prehabilitation (8, 9, 11, 21, 38). However a positive effect on pain following prehabilitation has previously been demonstrated (10, 12, 13, 15, 24, 38), which in some cases has even led to the surgery being cancelled or postponed (38, 39). The NP group also demonstrated lower joint awareness and higher quality of life compared with the P group both preoperatively and at 2 and 12 months’ follow-ups.

The present study demonstrated equal responses in both groups on the anchor questions provided to the patients at 12 months’ follow-up, both in self-perceived knee function and in willingness to have the surgery again. Taken together, this may be interpreted as both groups experiencing similar satisfaction 12 months following TKA. Some studies suggest an association between prehabilitation and reduced length of hospital stay; however, as hospital stay is influenced by multiple clinical and organizational factors, it remains a limited indicator of prehabilitation effectiveness, despite reported links with improved postoperative recovery (8–10). Some studies also suggest that prehabilitation may be associated with reduced postoperative complications, potentially through improved wound healing, inflammation control, and immune function (12). The present study demonstrated comparable lengths of hospital stay and similar complication rates, including equivalent proportions of readmissions and reoperations between the groups. These findings may be explained by the standardized fast-track care pathway applied to all patients in the present study.

Due to the lack of information on the patients’ functional state and grading of OA before attending physiotherapy in the present study, we do not know if preoperative physiotherapy increased the functional level in these patients until the preoperative time point. Baseline characteristics differed between the groups, with a higher proportion of women and more frequent use of walking aids in the P group. The observed differences between groups may therefore indicate that patients who opt for preoperative physiotherapy tend to have a lower functional physical level prior to surgery. This likely reflects a form of self-selection or selective referral, whereby individuals with more pronounced symptoms or mobility limitations are either more motivated – or more frequently encouraged – to participate in preoperative physiotherapy. Such baseline disparities underscore the importance of accounting for preoperative function when interpreting postoperative outcomes and were therefore adjusted for in the statistical analyses. Previous studies have shown that patients tend to resume their activity patterns after prosthetic surgeries (40), suggesting the P group may have been less active before preoperative physiotherapy.

There is no detailed information on the duration or the content of the prehabilitation other than that the patients received conventional preoperative physiotherapy. Likewise, no information is available on the postoperative rehabilitation, as to whether the patients had physiotherapy after the surgery. However, all patients were treated according to the fast-track clinical pathway with standardization of all parts in the treatment chain, ensuring equal information and follow-up for all patients. The main strength of the study lies in the large number of patients from a single hospital department where all patients received standardized treatment and follow-up. The patients also received the same preoperative information and were recommended to start physiotherapy as soon as possible after the hospitalization.

These results must be interpreted with caution. A key limitation of the study is the absence of reference measurements prior to the intervention period, which prevents assessment of whether the groups were comparable at the outset of physiotherapy. The observed descriptive differences in PROMs and pain scores between the groups may reflect selection bias rather than a true treatment effect. Patients in the NP group may have entered the study with a more favourable pre-intervention status, which could account for their superior outcomes both at baseline and throughout the follow-up period.

There is great variability in physiotherapy interventions and some studies show significant improvements with prehabilitation (10, 11, 13, 14, 16, 17, 24), while others do not (19–23, 38). Studies demonstrating significant improvements are mostly those with a focus on high-intensity strength exercises (10, 16, 17) which has been found to reduce pain, improve muscle strength, PROMs, and function preoperatively, resulting in a reduced length of hospital stay and faster recovery. Therefore, more emphasis might be placed on such studies focusing on high-intensity strength training to effectively improve outcomes after TKA. Future studies on prehabilitation interventions should consider clinical effectiveness, as distinguishing effective from non-effective interventions is crucial for patient outcomes and the use of healthcare resources (21).

In conclusion, after adjustment for baseline differences, no between-group differences in postoperative self-reported physical function were identified. The consistently inferior scores among patients who received preoperative physiotherapy across all time points may reflect systematic differences between patients who elect to attend physiotherapy preoperatively and those who do not.
